# Current place of probiotics for VAP

**DOI:** 10.1186/s13054-019-2325-9

**Published:** 2019-02-13

**Authors:** Harjeet Singh Virk, W. Joost Wiersinga

**Affiliations:** Department of Medicine, Division of Infectious Diseases, Center for Experimental and Molecular Medicine, Amsterdam Infection & Immunity Institute, Amsterdam University Medical Center, Academic Medical Center, University of Amsterdam, Meibergdreef 9, Room F0-117, Amsterdam, 1105 AZ The Netherlands

**Keywords:** Synbiotics, Probiotics, Ventilator-associated pneumonia (VAP), Gut microbiota, Randomised controlled trial (RCT)

With the advent of new technological advancements, our understanding of the gut microbes, their functionality and their roles in critical illness has advanced greatly. The microbiome of ICU patients is characterised by a loss of diversity, site specificity, microbial richness and overgrowth of pathogens, inclining towards a single taxon [1, 2]. Despite a lag in understanding behind application, an emerging number of studies now focus on the use of probiotics as offering promise to ICU patients for the prevention of antibiotic-associated diarrhoea, *Clostridium difficile* infections, multi-organ dysfunction, sepsis in neonates and—most notably—ventilator-associated pneumonia (VAP) [3–5].

VAP is an important cause of morbidity and mortality in mechanically ventilated patients and responsible for between 24 and 47% of ICU acquired infections [6]. Despite a plethora of existing VAP prevention strategies, results have been disappointing [7]. An emerging number of studies now focus on the use of probiotics for the prevention of VAP [3, 4]. However, despite optimistic results, some believe the growing interest in the microbiota has spawned an explanatory hype as the essence for understanding otherwise unexplainable phenomenon. Hence key questions require answers: Does it work? And if so, what is the mechanism? And most importantly, is it safe?

Does it work? A number of clinical trials and meta-analysis focusing on probiotics and critical illness, not least VAP, have been published over recent years [8]. A Cochrane review of probiotic therapy for VAP found a reduction in incidence, although evidence was of low quality [9]. The analysis of eight RCTs with a total 1083 participants showed that the use of probiotics decreased the incidence of VAP (odds ratio 0.70, 95% confidence interval 0.52 to 0.95, low-quality evidence) [9]. However, the aggregated results were uncertain for ICU mortality. Another more recent meta-analysis by Weng et al, from 13 RCTs (*n* = 1969) had similar findings. Again, they found no difference in length of ICU/hospital stay or duration of mechanical ventilation [7]. These meta-analyses suffer from inclusion of trials of low quality, significant between-study heterogeneity and—with the limited number of inclusions—failed to detect any publication bias which affects precision of findings [7]. Furthermore, the baseline incidence of VAP as well as the VAP definitions used can vary markedly between studies. Not least, there are large variations in probiotic strains used, dosing schema as well as route of administration.

Most recently, Shimizu et al. looked at the ability of synbiotics (that is a prebiotic plus a probiotic) to reduce complications in VAP and modulate gut microbiota [10]. The symbiotic used was a combination of *Bifidobacterium breve* strain Yakult, *Lactobacillus casei* strain Shirota and galactooligosaccharides. Among the 72 mechanically ventilated septic patients who completed the trial, the incidence of VAP was 14.3% in the synbiotics group versus 48.6% in the no-synbiotic group (*p* < 0.05) [10]. Unfortunately, the apparently lower incidence of VAP did not translate to a lower use of antibiotics, difference in bacteraemia, ventilator-free days or mortality. Regrettably, this study only managed to recruit 127 patients of the intended 150, over 5 years, due to slow recruitment of septic patients or exclusion of some patients who were on other probiotics [10]. Taken together, and despite these limitations, the aggregate of the currently available low-level evidence suggests an overall beneficial effect of the use of probiotics in the prevention of VAP.

What is the mechanism? If the protective effects of probiotics in the prevention of VAP are indeed confirmed in larger trials, it remains to be seen how the probiotics assert their protective effects. Described favourable effects of probiotics include induction of host cell antimicrobial peptides, release of antimicrobial factors, suppression of immune cell proliferation, stimulation of IgA production, antioxidative activity, inhibition of epithelial NFκB activation and other epithelial barrier protective effects (see Fig. 1) [11]. It is questionable if probiotics however are able to aid in the recovery of gut microbiota in critically ill patients. Of interest is a recent study that examined the in-depth effects of multi-strain probiotics on post-antibiotic reconstitution of the human mucosal microbiome that showed that probiotics induced a markedly delayed and persistently incomplete indigenous stool/mucosal microbiome reconstitution. In other words, in some conditions, probiotics can perturb rather than aid in microbiota recovery [1].

**Fig. 1:**
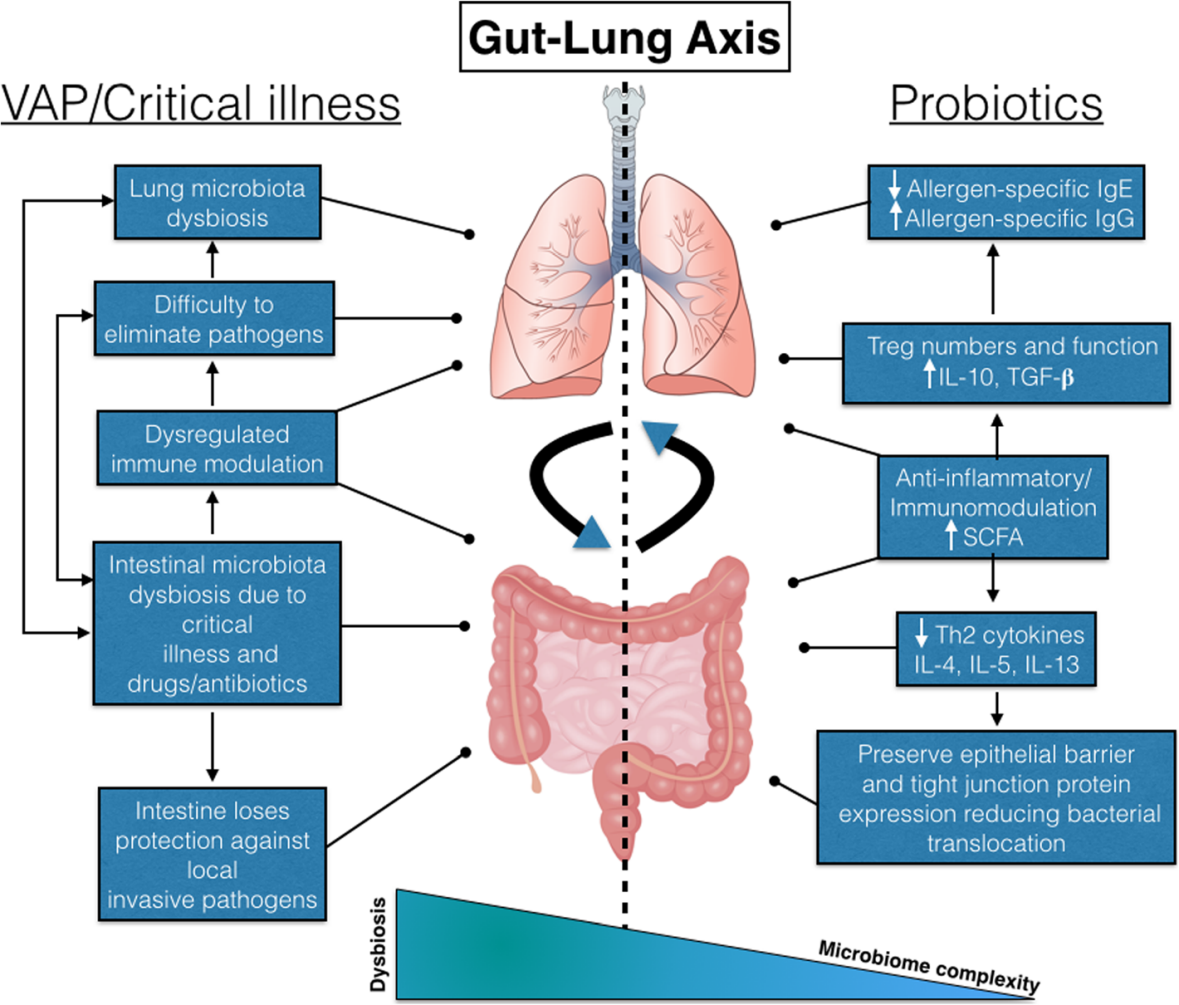
The gut-lung axis in ventilator-associated pneumonia (VAP) and proposed working mechanism of probiotics. IL interleukin, TGF-β transforming growth factor-β, SCFA short-chain fatty acids

The safety of using probiotics in critically ill patients has not been fully established. This has been a concern ever since the publication of the PROPATRIA trial, which—although criticised on multiple fronts—showed an increased mortality in patients with predicted severe acute pancreatitis on probiotic prophylaxis [12]. Resultantly, the Agency for Healthcare Research and Quality (AHRQ) commissioned a systematic review which concluded that although there does not appear to be an increased risk of adverse events with probiotic therapy in medium-risk and critically ill patients, reporting of adverse events is variable and current evidence does not provide specific answers to outstanding concerns of safety of probiotic therapy [13].

Many questions remain before confidence in rolling out probiotic or synbiotic treatment to critical care patients wide scale. Certainly, evidence suggest significant benefit for reducing VAP, but the question remains why? Perhaps answering this will open up avenues for better targeted therapy with reduced risk of side effects. Although prokaryotic lineages contribute the vast majority of the gut microbiome by abundance, important players are also potentially missed as the eukarya and viral microbiome remain incompletely charted. Furthermore, at what dose, and can a single probiotic be used in all populations and geographies? Our experience from past critical care trials proves this will be unlikely. Hence, we need more large-scale studies which can team up with specialists in microbiome research to analyse the mechanisms behind such outcomes.

It has been over 100 years since Metchnikoff first hypothesised that the heavy consumption of cultured yogurt by Belgian peasants may somehow account for their remarkable health and longevity [14]. Today, probiotics are big business with worldwide sales exceeding $30 billion [14]. Thus, the future of probiotics lies not only in supplementing beneficial functionalities, but also in affording the essential ecological context to sustain them [1]. Therefore, it is imperative that with increasing antimicrobial resistance and stagnating antibiotic pipelines, we nurture innovative research without compromising patient safety. We encourage standardisation of probiotic trials and reporting, together with enhanced fundamental research to avoid the perils of a one-size-fits-all approach.

## Abbreviations

AHRQ: Agency for Healthcare Research and Quality
FMT: Faecal microbiota transplantation
ICU: Intensive care unit
RCT: Randomised clinical trial
VAP: Ventilator-associated pneumonia

## References

[1] Suez J, Zmora N, Zilberman-Schapira G (2018). Post-antibiotic gut mucosal microbiome reconstitution is impaired by probiotics and improved by autologous FMT. Cell.

[2] Lankelma JM, van Vught LA, Belzer C (2017). Critically ill patients demonstrate large interpersonal variation in intestinal microbiota dysregulation: a pilot study. Intensive Care Med.

[3] Wischmeyer PE, McDonald D, Knight R (2016). Role of the microbiome, probiotics, and ‘dysbiosis therapy’ in critical illness. Curr Opin Crit Care.

[4] Haak BW, Prescott HC, Wiersinga WJ (2018). Therapeutic potential of the gut microbiota in the prevention and treatment of sepsis. Front Immunol.

[5] Schmidt TSB, Raes J, Bork P (2018). The human gut microbiome: from association to modulation. Cell.

[6] Grap MJ, Munro CL, Unoki T (2012). Ventilator-associated pneumonia: the potential critical role of emergency medicine in prevention. J Emerg Med.

[7] Weng H, Li JG, Mao Z (2017). Probiotics for preventing ventilator-associated pneumonia in mechanically ventilated patients: a meta-analysis with trial sequential analysis. Front Pharmacol.

[8] Manzanares W, Lemieux M, Langlois PL (2016). Probiotic and synbiotic therapy in critical illness: a systematic review and meta-analysis. Crit Care.

[9] Bo L, Li J, Tao T, et al. Probiotics for preventing ventilator-associated pneumonia. Cochrane Database Syst Rev. 2014;10:1–63. 10.1002/14651858.CD009066.pub2 PMC4283465 25344083

[10] Shimizu K, Yamada T, Ogura H (2018). Synbiotics modulate gut microbiota and reduce enteritis and ventilator-associated pneumonia in patients with sepsis: a randomized controlled trial. Crit Care.

[11] Klingensmith NJ, Coopersmith CM (2016). The gut as the motor of multiple organ dysfunction in critical illness. Crit Care Clin.

[12] Besselink MG, van Santvoort HC, Buskens E (2008). Probiotic prophylaxis in predicted severe acute pancreatitis: a randomised, double-blind, placebo-controlled trial. Lancet.

[13] Hempel S, Newberry S, Ruelaz A (2011). Safety of probiotics used to reduce risk and prevent or treat disease. Evid Rep Technol Assess (Full Rep).

[14] Morrow LE, Wischmeyer P (2017). Blurred lines: dysbiosis and probiotics in the ICU. Chest.

